# Damage of Splenic T Lymphocyte Proliferation and Differentiation and Its Normalization by Electroacupuncture in Morphine-Dependent Mice Mode

**DOI:** 10.1155/2011/424092

**Published:** 2011-06-09

**Authors:** Hong-Yu Li, Rong Zhang, Cai-Lian Cui, Ji-Sheng Han, Liu-Zhen Wu

**Affiliations:** ^1^Neuroscience Research Institute, Peking University, 38 Xueyuan Road, Beijing 100191, China; ^2^Department of Neurobiology, School of Basic Medical Sciences, Peking University, Beijing 100191, China; ^3^Key Lab for Neuroscience, The Ministry of Education, Beijing 100191, China; ^4^Key Lab for Neuroscience, The Ministry of Public Health, Beijing 100191, China

## Abstract

In a previous paper we reported that electroacupuncture (EA) could suppress opioid withdrawal syndrome and increase the appetite, sleep, and body weight in heroin addicts or morphine dependent animals. Considering that opioids were known to inhibit immune function, the present study was designed to observe whether EA could modulate the immune status of morphine dependent and withdrawal mice. We found that chronic morphine-induced decrease of splenic T lymphocyte proliferation and IL-2 production can be significantly raised by 2 Hz EA, and the fluctuation of CD4^+^/CD8^+^ ratio was also run to the baseline level by the EA. These findings indicated that chronic morphine exposure-induced immune dysfunction in mice could be normalized by 2 Hz EA.

## 1. Introduction

Chronic administration of opioid compounds can result in immune suppression [1, 2] and increased susceptibility to virus [3, 4] and bacteria [2] in heroin addicts. Several lines of evidence showed that acupuncture had positive immune modulation effects in human and animals [5–7]. Our previous studies showed that transcutaneous acupoint nerve electrical stimulation or electroacupuncture (EA) could ameliorate the withdrawal syndrome and increase the appetite, sleep, and body weight in heroin addicts [8] or opioid-dependent animals [9]. However, it is not unclear yet whether the EA could improve the immune function or not. In the present study, we observed the changes of splenic T lymphocyte proliferation, IL-2 production, and ratio of CD4^+^ and CD8^+^ T lymphocytes after chronic morphine treatment. We also explored whether above-mentioned changes could be reversed by EA treatment. 

## 2. Materials and Methods

### 2.1. Animals

All experiments were performed on male BALB/c mice from the Experimental Animal Center of the Academy of Military Medical Sciences, weighing 18–22 g at the beginning of the experiment. They were housed a single animal per cage in a 12 : 12 h light/dark cycle with food and water available at all times. The room temperature was maintained at 24 ± 1°C and relative humidity at 50%. Animals were conditioned and tested during the light phase of the cycle. They were handled daily during the first week after arrival. All experimental procedures were approved by the Animal Use Committee of Peking University Health Science Center.

### 2.2. Drugs and Reagents

Morphine hydrochloride (powder): Pharmaceutical Factory of Qinghai, China; concanavalin (Con  A) and [4,5-dimethylthiazol-2-yl]-2,5-diphenyltetrazolium (MTT3) were purchased from Sigma (MO, USA); IL-2 ELISA kit was purchased from Endogen (Rockford, IL, USA); RPMI-1640 culture medium from GIBCO (CA, USA); anti-CD4-phicoeritrin (PE), anti-CD8-isothiocyanate fluorescein (FITC), and isotype control from BD Pharmingen (CA, USA).

### 2.3. Apparatus

CO_2_ incubator was from Sanyo (Tokyo, Japan); 550 Enzyme Labelling Instrument and 1575 Micro bore Washing Machine from BIO-RAD (CA, USA); FACSCAN from BD (NJ, USA); Han's Acupoint Nerve Stimulator, HANS LH-800, was produced by Peking University of Astronautics and Aeronautics Aviation, Beijing, China.

### 2.4. Morphine Dependent Mice Model

**Figure 1:**
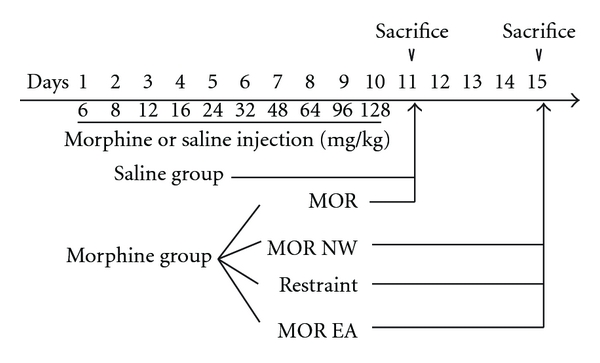
Experimental protocol. Forty mice were divided into two groups (saline and morphine) randomly. Thirty-two of them were injected with morphine with increasing dosage, and 8 mice were treated with saline in exactly the same manner. The morphine treated mice were further randomly divided into four groups: morphine control (MOR), morphine withdrawal (MOR NW), morphine plus restraint (Restraint), and morphine plus EA treatment (MOR+EA). *n* = 8 per group.

As shown in Figure 1, forty mice were divided into two groups (saline and morphine) randomly. Thirty-two of them were injected with morphine with increasing dosage, and 8 mice were treated with saline in exactly the same manner. Mice were given morphine with increasing doses from day 1 to day 10, Morphine was dissolved in saline and each animal was injected with morphine solution (0.1 mL/10 g body weight; s.c. t.i.d, 8 : 00, 14 : 00, 20 : 00). Eight mice were treated with saline in exactly the same manner. The morphine-treated mice were further divided into four groups. MOR: mice were treated with morphine from day 1 to day 10, and were sacrificed 8 h after last treatment. MOR NW: morphine nature withdrawal group, mice received no further treatment from day 11 to day 15, and were sacrificed on day 15. Restraint: mice were restrained in the plastic holders without acupuncture from day 11 to day 15, and sacrificed on day 15; MOR+EA: mice treated with morphine from day 1 to day 10, received electroacupuncture once a day from day 11 to 15, and then were sacrificed 2 h after the last electroacupuncture treatment (*n* = 8 per group).

### 2.5. Electroacupuncture Treatment

Mice were kept in specially designed holders, with their hind legs and tails exposed. Two stainless steel needles of 0.3 mm diameter were inserted into each hind leg, (+) electrode placed at the acupoint ST36 (2 mm lateral to the anterior tubercle of the tibia) and (−) electrode placed at SP6 (2 mm proximal to the medial malleolus, at the posterior border of the tibia). Mice's both hind legs were treated in the same manner, and then connected to the output channel of an electric pulse generator. Constant current square-wave electrical stimulation produced by a programmed pulse generator (HANS LH-800) was given via the two needles. The frequency of stimulation used was 2 Hz (0.6 ms pulse width). The intensity of the stimulation was increased stepwise from 0.7, 0.8 to 0.9 mA, with each step lasting for 10 min. The EA treatment was given once per day from day 11 to day 15.

### 2.6. In Vitro Lymphocyte Proliferation Assay

Mouse primary T lymphocytes suspension was prepared aseptically from the spleens of the mice. A single-cell suspension of spleen cells was obtained by pushing the spleen through nylon mesh bags in D-Hank's solution. The cells were collected and washed twice with D-Hank's solution (containing 2% FBS), centrifuged at 1000 rpm for 10 min, 4°C, and any remaining red blood cells were lysed by Tris-NH_4_Cl. The cells were suspended in RPMI 1640 medium, counted under the light microscope. The cell survival rate was found to be >90% with 0.2% trypan blue solution. The cell number was adjusted to 2  × 10^6^/mL, and 90 *μ*L cell suspension was transferred to each well of 96-well cell culture plate. 10 *μ*L ConA (5 *μ*g/mL) or RPMI 1640 medium was transferred to the well and made the final volume to 100 *μ*l. The cultures were incubated in a humidified 5% CO_2_ incubator at 37°C for 72 h. After incubation, 11 *μ*L MTT (5 mg/mL) was added to the each well of cell cultures and incubated for 4 h, and then 100 *μ*L lysis buffer was added. The plates were incubated overnight at 37°C and the OD measured at 570 nm.

### 2.7. Assay of Intracellular IL-2 Production

The single-cell suspension at 2 × 10^6^ cell/mL was prepared using methods mentioned above. After being cultured with ConA (5 *μ*g/mL) at 37°C in 5% CO_2_ incubator for 48 h, the supernatant was collected, and IL-2 content was measured with IL-2 ELISA kit according to the protocol of the manufacturer.

### 2.8. Splenic CD8^+^ and CD4^+^ T Lymphocytes Ratio Assay

The single-cell suspension at 1 × 10^7^ cell/mL was prepared using the method mentioned above. The cell suspension was transferred into three tubes, 100 *μ*L per tube: one was used as blank control, one incubated with mouse monoclonal anti-isotype, and the third tube was incubated with fluorochrome-conjugated anti-CD4 (phicoeritrin-PE) and anti-CD8 (isothiocyanate fluorescein-FITC) to double immunolabelled splenic CD8^+^ or CD4^+^ T lymphocytes. The samples were incubated at 4°C for 40 min, and washed twice with 0.01 M PBS buffer. The CD8^+^ and CD4^+^ T lymphocytes were resuspended with FACS protectant solution and analyzed, using a flow cytometer equipped with an argon laser and Cell Quest software.

### 2.9. Statistics

The results were expressed as the mean  ± S.E. and the statistical differences between various groups were determined by one-way analysis of variance (ANOVA), followed by the Newman-Keuls posttest (Prism 4.0). *P* < .05 was considered statistically significant.

## 3. Results

### 3.1. Effects of 2 Hz Electroacupuncture on Splenic T Lymphocyte Proliferation in Morphine Dependent and Withdrawal Mice

**Figure 2:**
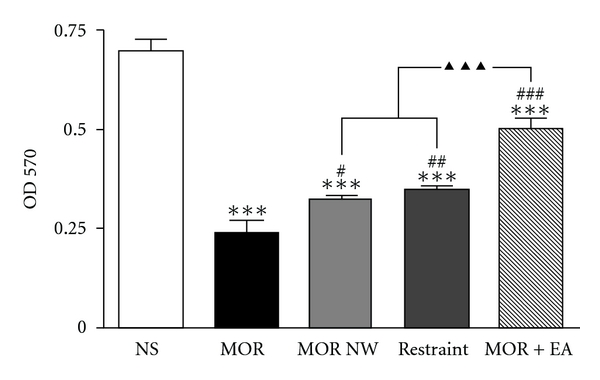
The effect of 2Hz electroacupuncture on the splenic T lymphocyte proliferation in morphine dependent and withdrawal mice. Data were shown as means ± S.E. and analyzed with one-way ANOVA followed by Newman-Keuls posttest. ***P < .001 versus NS group; ^#^ *P* < .05, ^##^ *P* < .01, ^###^ *P* < .001 versus MOR group; ^▴▴▴^ *P* < .001 versus MOR NW and restraint group. *n* = 6–8.

As shown in Figure 2, splenic T lymphocyte proliferation decreased to 34.5% of that of saline group 8 h after last morphine injection (morphine dependent phase) (*P* < .001), which recovered slightly 5 days after last morphine injection (morphine withdrawal phase) (*P* < .05), but still significantly lower than the normal level (*P* < .001). 12 to 16 h after last morphine injection, the mice were distributed into two groups, one was merely restrained in the holder for 30 min, serving as control for restraint stress, and the other received 2 Hz EA treatment 30 min per day for 5 consecutive days. It was shown in Figure 2 that the EA treatment accelerated the recovery of splenic T lymphocyte proliferation, compared with that of morphine dependent (*P* < .001) or withdrawal group (*P* < .001). However, the restraint *per se* had no effect on lymphocyte proliferation.

### 3.2. Effects of 2 Hz Electroacupuncture on Splenic T Lymphocyte IL-2 Production in Morphine Dependent and Withdrawal Mice

**Figure 3:**
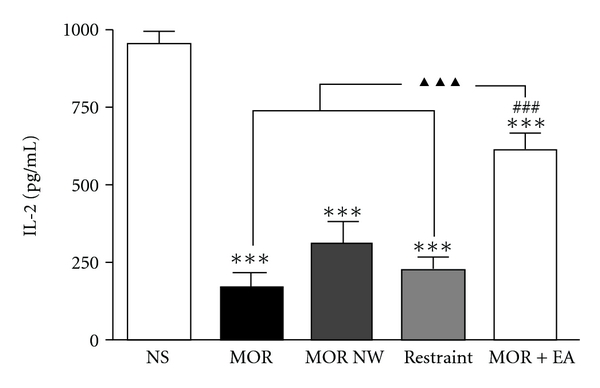
The effect of 2 Hz electroacupuncture on the Con A stimulated production of IL-2 in the supernatants of splenic T lymphocyte in morphine dependent and withdrawal mice. Data were shown as means ± S.E., analyzed with one-way ANOVA followed by Newman-Keuls posttest. ***P < .001 versus NS group; ^##^ *P* < .01 versus MOR NW group; ^▴▴▴^ *P* < .001 versus MOR and restraint group. *n* = 6–8.

As shown in Figure 3, the IL-2 content in the supernatant of splenic T lymphocyte culture of the morphine dependent mice decreased to 17.9% of that of saline control (*P* < .001). Five days after withdrawal the mice's IL-2 produced by splenic T lymphocyte was recovered to 33.6% of that of mice in saline group, but was still not significantly different from the morphine dependent group (*P* > .05). After EA treatment for 5 consecutive days, the IL-2 yielded by the splenic lymphocyte increased obviously and was significantly different from that of morphine dependent (*P* < .001) and withdrawal group (*P* < .01). Simple restraint was not effective in this regard.

### 3.3. Effects of 2 Hz Electroacupuncture on the Splenic T Lymphocyte CD4^+^/CD8^+^ Ratio in Morphine Dependent and Withdrawal Mice

**Figure 4:**
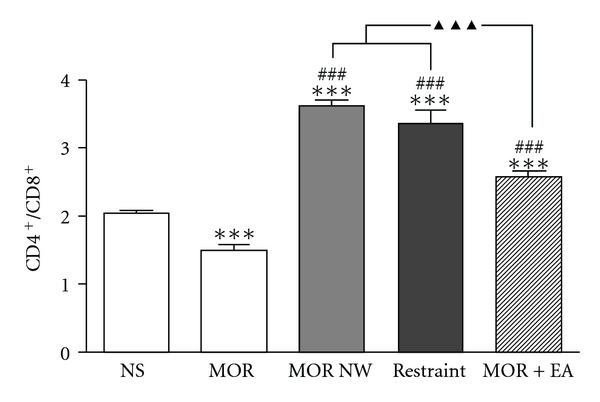
The effect of 2 Hz electroacupuncture on the ratio of CD4^+^ and CD8^+^ of splenic T lymphocyte in morphine dependent and withdrawal mice. Data were shown as means ± S.E, and analyzed with one-way ANOVA followed by Newman-Keuls posttest. ***P < .001 versus NS group; ^###^ *P* < .001 versus MOR group; ^▴▴▴^ *P* < .001 versus MOR NW and restraint group. *n* = 6–8.

As shown in Figure 4, 8 h after the last morphine injection, the splenic T lymphocyte CD4^+^/CD8^+^ ratio decreased slightly to 72.9% of the saline group (*P* < .001). Five days after morphine withdrawal, the CD4^+^/CD8^+^ ratio was not only higher than the normal level, but rebounded dramatically to a level of 175% higher than the saline control (*P* < .001). EA treatment for 5 days normalized the CD4^+^/CD8^+^ ratio to a level significantly lower than the morphine natural withdrawal mice (*P* < .001), and it was higher than that of morphine dependent mice (*P* < .001). Simple restraint was not effective in this regard.

## 4. Discussion

It has been reported that either acute exogenous morphine [10] or opioid receptor agonists [11] treatment could suppress lymphocyte proliferation and downregulate the function of immune system in animals. The mechanisms of effect above-mentioned morphine inhibiting immune system were mainly related with *μ* opiate receptors in the central nervous system (caudal region of periaqueductal gray) [12] but not in the periphery (i.e., on immunocytes) [13, 14]. On the contrary, systemic (intraperitoneal; i.p.) administration of [Met5] enkephalin with small dose (2.5 mg/kg) could increase the T lymphocyte proliferation [15], and selective *δ* opioid receptor antagonist, ICI-174,864 could block enhancement of T lymphocyte proliferation by [Met5] enkephalin analogs [16]. Therefore, consequences about the lymphocyte proliferation maybe related with the kinds of opioid receptors activated. In the present studies, chronic morphine administration for 14 days downregulated splenic lymphocyte production, which was similar with Carr's findings [17]. Carr and Carpenter reported that daily administration of morphine (50.0 mg/kg, s.c.) into alloimmunized mice (C57BL/6 into C3H/HeN) for 11 days resulted in a decrease in the number of thymocytes [17].

Acupuncture has been accepted as an alternative therapy in more and more people throughout the world. It is regard as a physiological homeostasis approach in regulating immune system have been investigated in healthy volunteers, suggesting that there was a statistically significant increase in the number of CD2^+^, CD4^+^, CD8^+^ CD11b^+^, CD16^+^, CD19^+^, and CD56^+^ cells as well as IL-4, IL-1*β*, and IFN-*γ* levels in the cells after acupuncture stimulation of meridian points [18]. Yu et al. found that acupuncture stimulation enhanced splenic natural killer cell cytotoxicity in rats [19], which may be realized through regulating IFN-gamma production [20]. Previous studies suggested that EA stimulation on “Zusanli" (ST 36) and “Lanwei" (Extra-37) points could prevent the decrease of lymphocyte proliferative response of rats induced by intrathecal injection morphine [21]. In the present study, low frequency (2 Hz), low-current (0.7–0.9 mA) EA stimulations and special acupoints (ST 36 and SP 6) were selected to use. 2 Hz represented low frequency, which was proved beneficial for endogenous enkephalin release [22]. In morphine dependent mice model, low-current EA was safer to avoid stress than high current, so we chose 0.7–0.9 mA current in practice. Zu-San-Li point (ST 36) was one of the special points regulating immune function as previously reported [23, 24]. We added San-Yin-Jiao (SP6) to constitute current circuit. All above parameters and acupoints in this study were selected seriously. It was shown that 2 Hz small current EA stimulation in ST 36 and SP6 reversed chronic morphine's suppressive effects on lymphocyte proliferation in mice (Figure 2). The result might be explained with the following mechanisms.

First, small-dose [Met5-] enkephalin could increase concanavalin A-stimulated proliferation of T cells in mice [15, 25], and our previous study has demonstrated that 2 Hz EA could accelerate the release of endogenous enkephalin in cerebrospinal fluid [22] and endogenous enkephalin might act on *δ* opioid receptor and then upregulate splenic lymphocyte proliferation.

Second, as we know, activated CD4^+^ lymphocyte can increase the production of IL-2, which can bind with IL-2 receptors to increase lymphocyte's proliferation [26, 27]. Morphine inhibited the transcription of IL-2 in activated human T lymphocytes [28]. We found in present study that 2Hz EA treatment for 5 days increased the IL-2 production (Figure 3), suggesting EA enhanced activities of CD4^+^ cells, and the latter yielded more IL-2 to accelerate lymphocyte's proliferation. 

In addiction, in physiological state, CD4^+^/CD8^+^ ratio is about 2. That is, the count of CD4^+^ T lymphocyte is about 60%, and CD8^+^ lymphocyte account for 30% of total lymphocyte. Both of higher and lower ratios indicate an abnormity of immune function [29]. In this study, CD4^+^/CD8^+^ ratio was downregulated in the morphine-dependence phase, but rebounded dramatically in the morphine withdrawal phase (Figure 4). We speculated that after morphine-withdrawal, the suppressed immunefunction is relieved, so the CD4^+^/CD8^+^ ratio rebounded. Nevertheless, CD4^+^/CD8^+^ ratio inversion or unconventional increase is abnormal. After 2 Hz EA treatment, CD4^+^/CD8^+^ ratio recovered to a relative normal range. Yamaguchi et al. reported that leukocyte cell counts appeared to return to appropriate levels after EA treatment in health volunteers [18]. We presumed EA played same role in morphine treated mice.

In the present study, we found that 2 Hz EA could improve splenic T lymphocyte proliferation and IL-2 production as well as a marked fluctuation of CD4^+^/CD8^+^ ratio in morphine dependent and withdrawal mice. We opine that role of the low-frequency EA on immune function as follows. (1) Zu-San-Li point (ST 36) was one of special points regulating immune function as previously reported [23, 24]. (2) The endogenous opioid peptides might be an important agent in the EA-induced immune regulation, but further investigation on the precise mechanisms of EA normalizing immune regulation in chronic morphine treated mice is requisite. In conclusion, our findings suggest that 2 Hz EA is a potential complementary therapy for improving immune dysfunction in opiate addicts.
